# Successful Transcatheter Arterial Embolisation for a Traumatic Iliacus Hematoma: A Case Report

**DOI:** 10.5704/MOJ.2003.016

**Published:** 2020-03

**Authors:** T Kato, N Chinzei, N Katayama, S Hirota, M Takahashi

**Affiliations:** 1 Department of Orthopaedic Surgery, Konan Hospital, Kobe, Japan; 2 Department of Radiology, Konan Hospital, Kobe, Japan

**Keywords:** traumatic iliacus hematoma, hip pain, transcatheter arterial embolisation

## Abstract

A traumatic iliacus hematoma is rare and usually occurs in patients after a fall involving a lower back injury. Although the hematoma may compress the femoral nerve causing femoral nerve palsy, the gold standard treatment for this condition has not been established. Here we report transcatheter arterial embolisation as a useful treatment strategy for a traumatic iliacus hematoma.

## Introduction

A traumatic iliacus hematoma is rare and usually occurs in patients after a fall involving a lower back injury^1^. The hematoma may compress the femoral nerve causing femoral nerve palsy, which results in severe pain and sensory disturbance along the distribution of the femoral nerve, and weakness or paralysis of the quadriceps muscle^1^. To date, the gold standard treatment for this condition has not been established, and reports in the available literature describing femoral nerve palsy secondary to a traumatic iliacus hematoma recommend operative or conservative treatments^1, 2^. Patel *et al* reported a similar case in terms of their patient’s age and the mechanism of traumatic iliacus hematoma treated by non-operative management^3^. A previous report has described successful angiographic embolisation before surgical treatment of the hematoma^2^. However, to our knowledge, no report has described transcatheter arterial embolisation (TAE) alone to treat a traumatic iliacus hematoma. Here we report TAE as a useful treatment strategy for a traumatic iliacus hematoma to prevent femoral nerve palsy.

## Case Report

A 14-year-old adolescent fell during a running high jump event and experienced left inguinal pain shortly thereafter. Within one hour after injury, she visited her orthopaedist, and although pelvic radiographs revealed no obvious fractures, she was unable to move because of persistent and in fact, worsening symptoms. Therefore, she presented to our hospital for further evaluation about two hours later after injury.

**Fig. 1:**
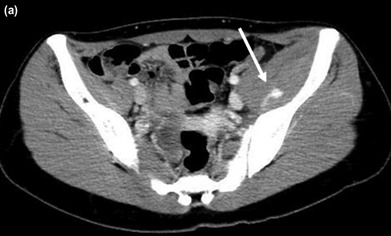
Contrast-enhanced computed tomography scan showing extravasation in the iliacus hematoma (white arrow).

**Fig. 2:**
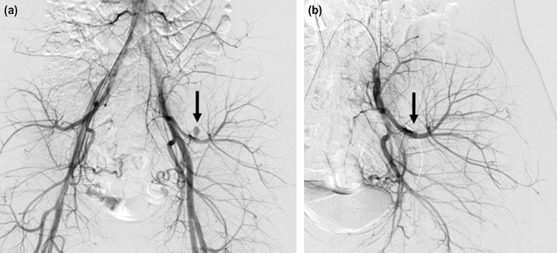
Left internal iliac arteriogram before and after transcatheter arterial embolisation (a) Left internal iliac arteriogram showing a pseudoaneurysm at the distal left iliolumbar artery (black arrow). (b) Left internal iliac arteriogram obtained after transcatheter arterial embolisation showing disappearance of the pseudoaneurysm (black arrow).

**Fig. 3:**
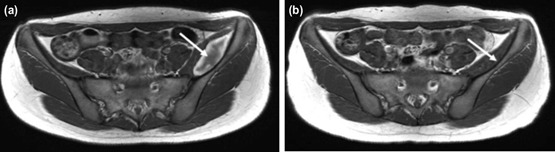
Magnetic resonance imaging scan showing the following findings: (a) Scan obtained 20 days post-injury showing an iliacus hematoma measuring 66×22mm in diameter (white arrow). (b) Scan obtained 80 days post-injury showing near-complete disappearance of the iliacus hematoma (white arrow).

Physical examination revealed tenderness over her left anterior and posterior hip. However, no obvious swelling was observed at these sites. She was unable to raise her left leg owing to severe pain; however, no sensory disturbances were observed in her left leg. Repeat radiographs were obtained to rule out obvious fractures of her pelvis and/or the left lower extremity. Because no displaced fractures were found, we still needed to exclude the possibility of fractures that could not be judged by radiographs. Furthermore, the patient’s symptom was so severe that she could not tolerate the long time taken for the magnetic resonance imaging (MRI) to be done. Therefore, we decided to diagnose by computed tomography (CT) for immediate further evaluation, which revealed edema of the left iliacus muscle (68×30mm in diameter). We performed a contrast-enhanced CT, which revealed extravasation of the contrast agent in her iliacus, indicating an iliacus hematoma with active arterial bleeding (Fig. 1). Thus, we performed TAE following consultation with our radiologists. Intra-operatively, we identified a pseudoaneurysm along a branch of the left obturator artery supplying the iliacus muscle (Fig. 2a) and embolised the pseudoaneurysm using a 25% mixture of n-butyl-2-cyanoacrylate [Histoacryl®, B. Braun Aesculap Japan, Tokyo, Japan] and iodized oil [Lipiodol®, Guerbet Japan, Tokyo, Japan] (Fig. 2b). After embolisation, we confirmed that the pseudoaneurysm disappeared, without any leakage of contrast agent from it. The patient’s symptoms gradually resolved following this treatment. She was placed on bed rest for 24 hours to ensure that the embolising agents remained at the treatment site, and postoperative rehabilitation was subsequently introduced based on her symptoms. Gait training was initiated 10 days after treatment. Magnetic resonance imaging (MRI) performed 20 days after treatment revealed a relatively large-sized iliacus hematoma measuring 66×22mm in diameter (Fig. 3a). MRI repeated 80 days after treatment revealed significant reduction in the size of the hematoma (Fig. 3b). No complications occurred until the latest follow-up (2 years), and she could resume sports activity. The patient and her parents provided consent to publish this case in a journal.

## Discussion

An iliacus hematoma can occur in patients receiving anticoagulation, as well as in those with hemophilia and liver cirrhosis, among other such conditions. A traumatic hematoma, as described in this case occurs following hyperextension of the hip or direct pelvic trauma. Usually, the femoral nerve passes through the psoas muscle and exits on the lateral aspect between the psoas and iliacus muscles in the pelvis^2^. Therefore, an iliacus hematoma can cause femoral nerve palsy by compressing the femoral nerve. Reportedly, conservative treatment can be considered in patients without progression of symptoms during follow-up^1^. In our case, no obvious femoral nerve palsy was observed during initial evaluation. However, we concluded that continuous extravasation in the hematoma predisposed the patient to a high risk of hemorrhagic shock and femoral nerve palsy. Therefore, we performed TAE in our patient. Left internal iliac arteriography revealed a pseudoaneurysm along the distal obturator artery, and we performed TAE to prevent adverse events. Rochman *et al* reported a case of femoral nerve palsy secondary to a traumatic pseudoaneurysm and iliacus hematoma in a 20-year-old man who showed complete nerve function recovery after angiographic embolisation of the pseudoaneurysm followed by surgical removal of the hematoma^2^. The authors concluded that embolisation could prevent possible rebleeding from the pseudoaneurysm.

In contrast, several other reports have described efficacy of conservative treatment for femoral nerve palsy secondary to a traumatic iliopsoas hematoma^1^. However, it should be noted that a few patients may develop late-onset femoral nerve palsy. A review of the literature revealed a study performed by Lefevre *et al* who reported clinical outcomes of femoral nerve palsy secondary to a post-traumatic iliopsoas hematoma in 16 patients^4^. Neurological symptoms developed in these patients approximately five days after injury. Of these 16 patients, 7 underwent conservative treatment and 9 underwent surgical treatment. All 9 patients with femoral nerve palsy who underwent surgical treatment showed complete neurological recovery. In the 7 patients who underwent conservative treatment, 6 showed complete neurological recovery and 1 showed incomplete recovery. The time required for neurological recovery ranged from 46 weeks in the conservative and 3-24 months in the surgical group. Notably, in patients presenting with femoral nerve palsy, it is unclear whether only embolisation of the bleeding artery can effectively treat this condition. However, this review indicates that once the patients develop femoral nerve palsy, they have the high possibility of undergoing surgical treatment, especially in some of whose neurological symptoms takes long time to recover. Based on these considerations, early diagnosis of an iliopsoas hematoma is essential to prevent femoral nerve palsy. Clinicians invariably encounter patients with groin pain with or without trauma and should be aware that iliacus hematomas must be considered in the differential diagnosis even in patients without radiographic evidence of obvious fractures.

From another viewpoint, if the patients undergo surgical intervention for hematoma removal, they must have bigger surgical scar compared to the one by TAE. TAE is less invasive and is associated with a lower risk of complications^5^. In the aforementioned study that described embolisation of the internal iliac artery in patients with pelvic trauma, no significant differences were observed in complications, such as skin necrosis, muscular atrophy, pelvic and perineal infection, nerve injury, and local pain between embolised and non-embolised patients^5^. In our case, her age was 14-years-old and we chose TAE as a less invasive treatment, not to ruin her precious adolescence by this traumatic iliacus hematoma and its complications such as femoral nerve palsy. Fortunately, her clinical course has been uneventful until the latest follow-up (2 years).

As a take home message, TAE is a useful treatment strategy for a traumatic iliacus hematoma. However, long-term follow-up of such patients is warranted to conclusively establish the efficacy of this treatment.
